# Topics of Public Interest

**Published:** 1918-04

**Authors:** 


### TOPICS OF PUBLIC INTEREST

**Attending Physician's Right to Fee as Expert Witness.** Birch vs. Lees, N. Y. Appellate Div., 165 N. Y., Supp. 846 and other cases cited, hold that the mere fact that a physician was an attendant and called to testify partly of questions of fact and ordinary observation, does not prevent his drawing a fee as an expert if asked to go beyond the statement of facts and to draw technical inferences and conclusions.

**Massed Psychology as a Military Asset.** Dr. John H. Quayle of Cleveland, at the annual meeting of Life Insurance Presidents, in December, called attention to the stimulating influence of the massing together of men in military cantonments, to the possibilities of using this influence for raising the standards not only of brute courage or even patriotism, but of mental strength generally, and of continuing this influence into times of peace. We are too prone to a pragmatic disregard of psychic power, sensibly directed and using the term itself in a sensible way instead of relegating it to cults founded on ignorance and superstition. The war arose from a mental process, it must be won by psychology directed along rational material lines. Pessimists have been alarmed at moral and especially venereal perils that they imagined would be increased because aggregated. They do not realize that a military exigency has not only called attention to physical defects but has remedied them, by operations of all sorts, by medical treatment and by sanitation and hygiene, not only inforced by authority but impressed on men who would otherwise never have learned the needed lessons. This course has followed the line of "one man at a time" but has been directed by a systematic study of large and small units. With an almost constant association of one medical man with an average clientele of less than 200, with the same association of an intelligent officer with about a tenth of this number and with every man, however ignorant, still more closely associated with his fellows, most of whom are intelligent enough to absorb the medical information constantly drilled into them, some two million young men from all parts of the country and all grades of the population have been raked over with the utmost care for weeks and months. In the aggregate, the constructive work along purely physical lines has been enormous. True, many cases are hopeless from the military standpoint but many are started on the road to physical health before being discharged and when discharged, the great majority have plainly put before them the means of physical reconstruction or of meeting incurable physical handicaps in the best possible way. The same information is available, from the military records, for public and private philanthropy.

The work done by the psychiatric board is, possibly, still more important in detecting essentially mental and neurotic conditions, in remedying them when possible, in calling attention to the necessity of some sort of guardianship in more severe cases. To a large degree, it has been demonstrated that unfitness for military service aside from gross physical and easily detected mental defects, is due either to neglected syphilis, resulting in central nervous lesions, or to essential psychic failure. Just as in the last few years, it has been shown that much of the evil of prostitution depends upon failure of mental development beyond the standards of childhood, so the psychiatric boards have proved that analogous evils in the conomic and social life of males—of course, directly reflected also upon the other sex—are equally important and probably depending in about the same proportion upon mental factors. The excuse offered by many for not enlisting, that the war should be fought by those out of work and without responsibilities, has been most emphatically rebutted by the experience of these boards. The men recommended for S. C. D. proceedings (discharged on Surgeon's Certificates of Disability) are, barring gross physical defects which should never have escaped examining boards, very largely those who have been unable to hold their jobs by reason of inherent and unrealized mental defects.

But, before reaching the stage of actual physical or mental deficiency, we have the men with a streak of yellow in them, the thoughtless, ignorant and selfish, the unpatriotic, the shirks. These men are poor soldiers and bad citizens, they are even to a large degree in bad health or liable to become so simply as the result of bad mental self control, yet they are not absolutely defective either physically or mentally, from the military standpoint. Held under military discipline for a few weeks or months, what happens? Before answering this question, two facts should be distinctly understood. Military discipline now means something much broader than most of us have understood the term. It means that every influence for physical, mental and moral health is placed about the soldier and that every influence and authority is exerted to impress these influences on him. When we say **mean**, we **mean mean.** Those who think that Uncle Sam is four-flushing in commissioning chaplains and recording the religious preferences of soldiers on his official blanks, in giving place to the Y. M. C. A., K. C., Red Cross, in restricting leaves of officers and men, in commanding abstinence from alcoholic liquors, in inviting prostitutes to leave the vicinity of camps, are mistaken. The other fact is that the optimists who held that the general average of morals, patriotism and intelligence of our young men was reasonably high, are right. Thus, we get an idea of what Dr. Quayle implies in his term "massed psychology." The tendency is to eliminate the waste products of faulty psychic metabolism, to clear out a mass of uncleanness and to bring about a state of psychic health, exactly as happens in purely physical ways under the hygienic, sanitary and curative direction of the medical corps. Of course, as in physical conditions, there is a residuum of the hopelessly depraved. This is eliminated gradually for the man who wont be a good soldier is ultimately certain of securing his discharge—though not necessarily with the freedom for pursuing his low inclination in the safety of civil life which he craves. We may be appalled at knowing statistically the percentage of physically defective young men in the country, at knowing just what percentage is mentally deficient, at having dumped back from the army, the cowards and weaklings but we are far safer to have all these classes segregated, at least potentially.

---

**Food Prices.** The State Commission has published wholesale and retail prices, the former more or less subject to regulation, the latter not susceptible of control except by the general regulation of profits introduced as a war measure but proposed as a guide to consumers. While radicals may hold that absolute fixation of prices should have been legalized, it should be remembered that the gradual rise in prices before the war was due largely to false pride which prevented purchasers from applying to foods the same business-like bargaining which they practiced for certain other necessities of life, notably rentals. Thus, in putting it up to the ultimate consumer to insist that the retailer should hold the retailer to a fair profit, and should stand between the consumer and the wholesaler in a determination to hold down prices, the best leverage for permanent results is provided. The prices proposed render it possible, by using wheat and the cheaper cereal products for half or three quarters of the ration, by limiting meats to the provision of the optimum minimum, by avoiding large quantities of innutritious vegetables, still relatively cheap by the pound, but never economic in proportion to their food value, and by a judicious selection of fruits, to set a very appetizing though plain table at 20-25 cents a day.

---

**Conservation of Philanthropic Effort.** We have several times alluded to the enormous waste of effort and money by the haphazard undertaking of all sorts of worthy causes by all sorts of private organizations, in time of peace. The stand taken by the government authorities in regard to concentration of certain lines of private philanthropy in the Red Cross is well known and while it is disappointing to some, the wisdom of the course is beyond dispute. One of the strangest campaigns is that undertaken by an organization of young women, to secure funds to enable them to stamp out the venereal peril. Just how any campaign with this end in view, is necessary to supplement the military efforts, which are as efficient and as practical as can be devised, is an open question—unless indeed one to force negligent civil authorities to carry out the letter and spirit of existing laws.

It is especially difficult to explain the method on which a number of young women would actually carry out such a campaign. The only explanations that we have heard are too cynical to repeat.

**Caloric Value of Wood.** In view of the propaganda to save coal by burning wood, whenever available, it is interesting to note that hickory, oak, beech, birch, hard maple, ash, locust and long leaf pine (containing much resin) weigh about 2 tons per cord and have a caloric value of about half that of coal. Short-leaf pine (not so resinous), hemlock, sycamore and soft maple weigh 1½ tons per cord and about 1½ cords are required to equal a ton of coal. Cedar, redwood, poplar, cypress, catalpa, Norway pine, spruce, and white pine weigh about 1 ton per cord but 2 cords are required to equal a ton of coal. Dry wood is, of course, richer in calories than moist, unless the "moisture" consists of resin. As a general rule, weight for weight, most woods have about half the heating power of coal.

**Income Tax Statistics 1916.**

| | | Per Mille |
|---|---|---|
| Over five million | 10 | |
| One to five million | 196 | |
| 200,000 to 1 million | 2,238 | 0.25 (Over five million to 100,000) |
| 150,000 to 200,000 | 1,284 | |
| 100,000 to 150,000 | 2,900 | |
| 50,000 to 100,000 | 10,452 | |
| 15,000 to 50,000 | 59,311 | 4.6 (50,000 to 10,000) |
| 10,000 to 15,000 | 45,305 | |
| 5,000 to 10,000 | 150,551 | 6. |
| 4,000 to 5,000 | 72,027 | 6.3 |
| 3,000 to 4,000 | 85,122 | 6.3 |
| (Discrepancy noted) | 437,036 | 17.4 |

355,107 of the payers are married men; 47,461 single men; 26,833 single women; 7,635 married women making separate report. The average family consists of about 5 members and it may be assumed that the income of the head of the family fixes the financial and sociologic status of the whole family. However, the additional incomes reported make it necessary to assume that, in the financial sense, the average family consists of only about 4 persons. This corresponds quite accurately with the priori probabilities based on age, sex and incapacity, that about 25% of the population consists of wage-earners or responsible sources of family income and the column of per mille distribution of wealth is computed on this basis, namely of 25 million average families from the standpoint of source of income. According to these statistics, rather less than 2% of the whole population can be considered as even well-to-do—and note that we are dealing with average family groups, not with individuals—or can maintain the standard of living, philanthropy, automobile upkeep, travel, generally accepted as "normal." There is a discrepancy somewhere.

---

**Buffalo Water Supply.** After checking up the waste of water, Buffalo having enjoyed for many years the record of having the most liberal—or, according to some, the most exaggerated per capita water supply of any city in the world—it has been shown: 1. that either the recent 1-million-dollar intake system or the older one is adequate; 2. that, under ordinary conditions, the water is practically the same whether it flows through a pipe from the end of Lake Erie or in its natural middle channel in Niagara River to the former intake; 3. that, while under abnormal conditions, as disturbance of currents by winds (acting by changing levels rather than by actual blowing of water) the more recent method affords dangerous or suspicious water less frequently and for shorter periods, it does not even in the practical sense afford an adequate guarantee of purity. The chlorine method has given quite satisfactory results but is not ideal from any standpoint and, apparently the only radical solution of the problem is to institute a sand filter somewhere, there being neither a cheap and convenient source of sand nor a cheap, unoccupied place upon which to establish a filter. In short, we have wasted 7 million dollars, representing dead capital investment of about $15 per capita—men, women and children—on a temporary expedient which has not even temporarily obviated the necessity of additional measures toward water purification and which has, at most, afforded insurance against failure of supply from conceivable causes which have not materialized. Possibly, by universal antityphoid vaccination, we may ignore minor impurities and be contented with a fairly good natural water. At present, sanitarians are loath to subscribe to such a course. If we accept the general view that sand filtration is the only efficient method, we are compelled to face engineering problems of great magnitude and expense. So far as we are aware, the following are the main solutions proposed: 1. Ignore Lake Erie altogether, secure an entirely different water supply from surface drainage, at a distance, which will reach the city without pumping on account of original higher level, but which will involve enormous expense for drainage area, impounding and conveyance by pipe lines or aqueduct;

2. Artesian wells; 3. Secure at practically no cost for site but enormous cost for preparation a sedimentation and filtering area in the upper part of Niagara River; 4. Condemn land expensive on account of improvements, at or near the present pumping station or reservoirs; 5. Establish new reservoirs or subsidiary pumping stations at a considerable distance from the present ones but somewhere within or near the present city limits. On general principles, it might be suggested that the first alternative could be modified by sacrificing water head, saving distance of conveyance and pumping from a nearer area of drainage and impounding but there does not seem to be any such site which is not occupied by villages of such magnitude that the expense would far exceed the first alternative as stated. We trust that the problem will receive the attention of the medical profession. It is exceedingly complicated and requires long study from all aspects.

---

**Psychiatry—Its Military Importance.** In the British army, it is said that the discharges for wounds and for disease are about equal and that neurasthenia, including shell shock, amounts to 8% of the latter. In the first 2½ years of the war, 8,000 cases of epilepsy were discovered—approximately 2:1000. While definite statistics are not available and probably will not be till after the war, it is safe to say that this subdivision of neurology has developed an importance not previously realized by the medical profession in general. Cases of deficient mentality, hysteria, etc., have, of course, passed ordinary examining boards while more marked physical defects and such diseases as tuberculosis in moderately advanced stages, have been excluded at the outset. It is not at all surprising that the strain even of camp life and especially nostalgia should bring cases of essential mental deficiency to the attention of company commanders but it is surprising to many complacent physicians to find that the discharges for this reason exceed those for incipient—and possibly exacerbated—tuberculosis, venereal disease, gross physical disability, etc. Another surprise is that quite a number of men of undeveloped mentality have attained the rank of corporal or sergeant or even have received commissions. Quite well marked insanity has also manifested itself in quite a number of cases, though comprising but a small fraction of a per cent of all troops. The unfortunate experience with these hitherto unnoticed cases, in mobilization camps, arouses wonder that the Germans should have experimented with the use of persons known to be insane as combatants. We can heartily agree with the German dictum that they are a nuisance at the front. The psychiatric boards have called attention to the gradation from actual malingering through all sorts of hysterical manifestations in which the neurotic manifestations are not even indirectly dependent on this element. In one instance, a man had deliberately concealed the fact that he had been in an insane asylum, so that he might enlist. His conduct had been so good that he had been made a sergeant and his condition of dementia precox had been discovered only after he had been sent to the hospital for an organic disease of quite different type. Many a normal individual should take his hat off to so heroic a defective.

---

**Variola and Race Discrimination.** It is noteworthy from a legal standpoint, in view of the constitutional provisions against race discrimination, that the N. Tonawanda Board of Health has ordered all negroes to report for vaccination or leave town. The order is justified for the practical reason that 39 cases have been reported in Buffalo, 2 in Niagara Falls and 1 in La Salle (up to Dec. 1) and all among negroes.

---

**War Activities in Regard to Tuberculosis.** The Bulletin of the National Assn. for the Study and Prevention of Tuberculosis, suggests some excellent though obvious methods of education. We rather question, however, the wisdom of attempting to educate the soldier, including the medical military officer, through non-official channels, such as the Y. M. C. A., K. of C. or moving-picture entertainments, unless assistance is directly requested by the authorities along definite lines and in particular instances. The government is, officially, putting into execution the most elaborate and practical system for dealing with the tuberculosis problem.

---

**Alfalfa Flour,** cooked in various forms about as wheat flour and alfalfa candy made from glucose prepared from the stalks, has been made available. These preparations are said to be palatable but this is no time for fooling ourselves by false economies, physiologic or financial and we trust that thorough, practical tests will be conducted to establish nutritive value and that the price will be set so as to conform to the average for cereal preparations generally.

---

**Negroes in the Medical Reserve Corps.** 30 were appointed about Jan. 1.

---

**Mental Defectives** discovered by psychiatric boards of the army, amount to about 1% of the total passing physical examinations. The practical impossibility of discovering and rejecting these men at the initial examination is illustrated by the fact that quite a number have become non-commissioned officers and a few have received commissions. A good many of the defectives are highly patriotic and make good soldiers until a certain degree of strain is reached, including stration of the psychiatric boards is that the defectives are that of monotony of service. One immensely valuable demon-largely made up of men who have not held their jobs. The plea that the army should be drafted from men out of work is thus negatived. Perhaps even more important is the hint as to the correction of the economic evil of lack of employment in peace.

---

**Berlin Food Prices.** Butter, $2.25 a pound, sugar 56 cents, ham and bacon $2.11. American soap sells at about 22 cents a bar.

---

**Centenarians.** David Mosher of E. Aurora, died Nov. 18, 1917, aged 100 years, 7 months and 22 days. Mrs. Samantha Nellis of Naples, born in Herkimer Co., N. Y., celebrated her 100th birthday Jan. 8, 1918. She is in good health. Mrs. Hannah Gibson Leslie of Jamestown, widow, celebrated her 100th birthday Dec. 31, 1917. Mrs. Mary Wright of Buffalo, whose will in 1893 stated that she was sick and about to die, survived till Sept. 22, 1917, when she did die at the age of 107.

---

**Triple Typhoid Vaccination** is asserted by the National Anti-Vivisectionist Federation to be responsible for death and disease in soldiers at cantonments and the blame is placed on Surgeon-General Gorgas. Or rather, serum inoculations are stated in general terms to be the cause. So far as we have learned, triple typhoid and anti-variolar vaccination are the only procedures to which the more general term can be applied. Other procedures, such as the use of antimeningococcus serum, diphtheria antitoxin, etc., have been applied only when the corresponding diseases have developed, and the mortality has been lower than in older series without such treatment. There have been a few cases of quite sharp reaction to the prophylactic vaccinations universally practiced, but so far as we have learned, no fatalities or even alarming cases. Typhoid has occurred in small proportionate incidence and in mild form with low mortality, mainly at the beginning of cantonment mobilization, i.e. introduced with the troops. In some cantonments the typhoid wards have been empty after the first few weeks. Smallpox has been almost nonexistent. The only diseases which have occurred with marked frequency have been pneumonia, ordinary colds (infectious, undoubtedly), measles including German measles, venereal diseases, which have shown a marked decline as time has elapsed, and which probably not even the antis would ascribe to the prophylactic measures against smallpox and typhoid and the para-typhoids, and, in a sense, meningitis. We say "in a sense" because the ideal of the Medical Service is to eliminate this disease. It has not developed to the extent formerly taken as a matter of course, and the mortality and paralytic developments have been low. Even if there were any reason to assume that anti-variolar and triple typhoid vaccination were in any way responsible for measles, pneumonia, colds and meningitis, the balance between the mortality from all these diseases together, and the probable mortality from smallpox and typhoid without vaccination is in favor of the continuance of the prophylaxis. One point that seems to have been generally overlooked is the childishness of regarding these vaccinations as serious procedures.

---

**Dogs.** The state system of licensure has shown that taxes were paid on 282,243 dogs last year. Only 950 claims of damages were made, amounting to $77,862. According to the ordinary ratio between claimed and actual damage, it may be estimated that the average dog did about 5 cents worth a year. 2,951 sheep were reported killed and 12,692 injured or worried. When, without hearing the other side of the question, it is claimed that there was one sheep killed for approximately 100 dogs, the exaggeration of this danger is apparent. It is difficult to estimate how many sheeps' lives were directly or indirectly saved by dogs, or how much human labor was saved in herding sheep and cows but the balance is obviously to the credit of the dogs by a wide margin. On a farm carrying 100 sheep and 10 cattle a good shepherd dog—not necessarily of the breed thus known—will easily save a third of a man's time. Some sort of statistics ought to be gathered of the saving of human life and property by watch dogs.

---

**Weekly Death Rates.** The Bureau of the Census now issues sheets giving the deaths and rates for all principal cities of the country by weeks. On account of marked changes in population, not even subject to check by estimates, we do not attempt to publish these statistics but suggest that anyone especially interested should apply for these reports.

---

**Mortality Statistics, 1916, Registration Area.** This area now includes about 70% of the population and the total number of deaths was slightly over a million, about 14:1,000 population. Tuberculosis fluctuated about 2:1,000 until 1904 and has gradually and apparently genuinely declined to 1.416:1,000. Pneumonia shows marked fluctuations, averaging a little lower than tuberculosis. Bright's disease, cancer, diabetes, apoplexy and notably arterial diseases 6.1 in 1900 to 23.9:1,000 in 1916, have increased, probably partly due to gradually increasing age, largely to refinement in diagnosis, although the last disease is rather a matter of conception and nomenclature than actual detection of a condition. Diphtheria has declined from 0.433 in 1900 to 0.145:1,000. Typhoid from 0.359 to 0.133. Measles, pertussis, scarlet fever, etc., fluctuate considerably but it is worth remembering that, with the exception of ''influenza'' none of the ordinary acute infections reaches a mortality rate of 0.15:1,000. Just about 6% of all deaths are by some form of violence. Those due to railroads run about 10 per hundred thousand population; those due to street cars about 2.5, a gradual decline, while those due to automobiles have increased from 0.4 in 1906—the first year for which they were separately recorded—to 7.3 in 1916.

---

**Salvarsan** patents have been abrogated by the government and firms will be licensed to prepare this drug under the name of arsphenamine, subject to rate control. From an ante-bellum price of $4 per dose, up to a rate of $35 due to inadequate supply, the price will now drop to $1 for the army and navy, $1.25 for hospitals and $1.50 for physicians and it is asserted that an abundance will be almost immediately available.

---

**U. S. Victims of Shell Shock Have Arrived.** The first U. S. victims suffering from shell shock and mental disorders have been brought to Fort Porter, Buffalo, and to Fort Niagara, Youngstown. These sufferers will be given the same course of treatment in use abroad.

---

**Glens Falls** is the first of the third-class cities of the state to have a full-time health officer, Dr. Virgil D. Selleck, at a salary of $3000. It also maintains a public health nurse and a tuberculosis dispensary. The population is a little less than 17,000.

---

**Typhoid Fever has Declined** in N. Y. State from a little over 30 deaths per 100,000 population to 5.8 in 1917. For the last 10 years the average was 11, for the 17 years from 1885 to 1907, 23.

**Accidental Poisoning** kills about 5000 persons in this country, annually, according to the Surgeon General of the Army. A special bottle is suggested, with skull and cross bones molded in the glass so that they can be felt in the dark, is suggested.

---

**Military and Preventable Deaths.** Some one has calculated that 7 million have been killed in the present war, in all the countries engaged and that 6½ million die in the same countries from preventable causes every year.

---

**A Universal Reserve Corps.** The matter is being seriously considered of making every physician a member of a reserve of the reserve corps. Potentially he is so already just as every citizen is potentially liable to compulsory service in one way or another.

---

**Premier Georges Clemenceau** of France, born in 1841, was a physician and the son of a physician. He practiced in N. Y. City between 1865 and 1869 and is also said to have practiced in Stamford, Ct. It is said that he failed in his professional career, but so does everyone who does not continue in it. The same statement was made of the late D. Hays Agnew of Philadelphia, who left medicine for business early in life but later returned to his practice and became one of the world's most noted surgeons.

---

**Increased Rank for Medical Corps and Medical Reserve Corps of Army.** Senator Owen's bill has been reintroduced, Representative Dyer introducing the bill in the House. In its amended form, the bill follows the provisions for the Navy, decreasing the lieutenants and captains from 67.2 to 64%, majors from 23.7 to 23.5%, increasing lt. colonels from 5.42 to 8%, colonels from 3.16 to 4% and providing for 0.5% brigadier generals. The ranks beyond major are opened to the reserve also. It seems to us, that merely from the standpoint of efficiency, it is a good plan not to have any large number of men feel that they have no further advancement in prospect.

---

**Some Modern Military Statistics.** Bayonet wounds, in the Civil, Balkan and present war, have usually proved fatal so that they figure to a small degree in the statistics of wounds treated (about ½% in the Civil War). In the Civil War, about 8% of wounds were due to artillery, 92% to rifle bullets. In the Balkan War, artillery wounds comprised about 25% and this percentage is still larger at present, though exact general statistics are not available. Not more than 5% of wounds at present are typic bullet wounds. 64% mortality in penetrating abdominal wounds is the best rate thus far secured.

---

**Good Roads.** The N. Y. State appropriation for this year is cut from $5,400,000 to $3,787,383, to cover repairs, maintenance and construction. This provides for not much more than a hundred miles of hard surface roads, and let us hope that no more money will be wasted on grease and flint highways. One hundred miles of new work, plus whatever can be paid from licenses will mean very little if fairly distributed, much if planned so as to supply missing links.

---

**Army Medical Service.** Feb. 23, the medical corps of the regular army numbered 768, the reserve 13,687, the national guard 1,207, the national army 32, total 15,694. 1,050 have been discharged from the reserve corps, 411 for physical disability, 154 for inaptitude for service, 306 to serve the government in other ways, 59 on account of domestic difficulties, probably mainly of financial nature or because of special need of personal attention to business matters, care of dependents, etc., 88 by resignation without specification of reason, 32 on the ground that they were needed by communities, hospitals, colleges, etc.

The regular army corps (768) includes 1 major general (Surgeon General Gorgas), 61 colonels, 103 lt. colonels, 210 majors, 2 captains, 391 lieutenants.

The reserve corps (17,052) includes 1,011 majors, (the highest rank to which eligibility yet exists), 3,762 captains, 12,279 lieutenants. Of these the 13,687 called to service, include 844 majors, 3,227 captains, 9,616 lieutenants.

The national army corps (32) includes 4 brigadier generals, 10 colonels, 16 lt. colonels and 2 majors. It is generally understood that, so far as the distribution of the various branches of the service is concerned, there is practically no limitation to troops of corresponding designation, except that the national guard surgeons remain, for the most part, with the organizations representing those with which they were originally connected. Thus far, the national army medical corps has been utilized for the promotion of men of the regular or reserve corps, to grades for which there are no vacancies in the former and no provision in the latter, in cases in which service beyond the period of the war is not contemplated.

---

**The Medical Reserve Corps.** Without in any way desiring to detract from the glowing accounts of some writers, volunteers should realize that we are at war and that, even in this country, this means a sacrifice of the comparative leisure and luxury properly enjoyed in the regular army in peace, with small numbers, adequate posts and a perfectly working routine without need of haste, by men gradually trained to the maximum of efficiency. It is not simply a matter of extra work and temporary discomfort due to an accidental cause. Much of the extra work is in preparation for actual emergencies that may be expected in Europe and much of the lack of minor conveniences and comforts, while not deliberately planned by the authorities to hamper new men in their work, is viewed philosophically, as a means of testing physical and mental strength, developing resourcefulness and that combination of initiative and dependence on authority that is necessary in the training of any soldier. For example, there is often compulsory attendance on clinics and lectures, and presentation of the same in rotation, occupying two or three hours a day, in addition to obviously necessary duties and without regard to personal preference and interest. Sometimes this really means a considerable strain. Detail as officer of the day involves the fatigue of regular and manifold special duties for long hours, indeed, with only so much sleep as a fortunate absence of emergency night calls allows. What you have read in the Regulations concerning the number of rooms or cash allowance when not provided, allowance for light, fuel, etc., privilege of buying from the quartermaster, assistance in keeping a ''mount,'' orderlies, ''strikers'' (not of course so definitely described in any official document), transportation of baggage, leave of absence on full and half pay, etc., etc., etc., is very largely a dead letter in time of war. You can count on your salary, enough room in a tent or some sort of barracks for your cot and your clothes, a moderate minimum of heat, light, etc., rather crude conveniences in the way of plumbing or what preceded plumbing in the last century, office equipment, etc. The only perquisites that you may expect are your first travel allowence which just about doubles your actual traveling expenses and very few repetitions of opportunities for travel without troops. If you make good, and the government really needs to train you along special lines, you may enjoy a splendid postgraduate course at government expense but it is by no means a sinecure. You have got to learn to do efficient medical work in spite of noise and confusion, perhaps in the midst of construction work, and in spite of untrained and shifting, often numerically inadequate assistance. You will have to watch your chance to get a typewriter (machine, not persons)

and will have to be your own interne and clerk and perhaps to write on a packing box and drive a nail to hold your hat. On the other hand, you will meet with a few hardships that you cannot readily excuse in view of the rapid need of establishing ways and means to a large amount of work and none that you cannot regard as either liable to be duplicated in the actual field of hostilities or, at least, as in some way preparing you for service at the front. On the other hand, so far as our experience goes, you will find no reasonable request refused for facilitating your work or securing your own proper enjoyment and maintaining your physical and mental well being — with due allowance for established methods of doing business and factors over which no one has control. You will find no hardship or fatigue not exceeded by enlisted men or even line officers, no executive difficulties and demands for adaptation in the face of all sorts of obstacles which your superiors in the medical corps are not meeting cheerfully.

---

**Improvement in Juvenile Delinquency.** As against the increase in prevalence of crime and misdemeanors by children in many of the beligerent countries, it is interesting to note that the children's court of N. Y. City showed a falling off from 7,927 cases in 1915 to 5,970 in 1916. Low immigration, higher wages of parents, quarantine on account of the meningitis epidemic, are given as the chief causes.

---

**Sexual Prevalence of Suicide.** Suicide is rare in childhood, though by no means unknown. In the adolescent period, 15-19, the rate is about 11: 100,000 persons living, for both sexes. In females, the rate then remains nearly stationary but for males, it gradually increases to the maximum of slightly over 80: 100,000 for the age group 65-74.

---

**The "Venereal Menace of the Army."** There seems to be no fallacy in these statistics: From Sept. to the last week of Dec., 1917, the incidence of venereal disease kept rather close to 80: 1000 per year, though one weekly rate was 51 and one 165. This may, in a sense be considered the normal average for soldiers. The National Guard showed an incidence varying at first from about 140 to 150, falling to about 45. The National Army, from an incidence of 387 for the second week—the first week's incidence being put at 193 but the marked increase being obviously due to lack of time to discover cases—to about 67 for the last two weeks. These statistics show that it is not army life but civilian that is a menace from the venereal standpoint.

